# Tranilast Increases Vasodilator Response to Acetylcholine in Rat Mesenteric Resistance Arteries through Increased EDHF Participation

**DOI:** 10.1371/journal.pone.0100356

**Published:** 2014-07-03

**Authors:** Fabiano E. Xavier, Javier Blanco-Rivero, Esther Sastre, Laura Caracuel, María Callejo, Gloria Balfagón

**Affiliations:** 1 Departamento de Fisiología, Facultad de Medicina, Universidad Autónoma de Madrid, Madrid, Spain; 2 Instituto de Investigación Sanitaria IdIPaz, Madrid, Spain; 3 Departamento de Fisiologia e Farmacologia, Centro de Ciências Biológicas, Universidade Federal de Pernambuco, Recife, Brazil; University of Southampton, United Kingdom

## Abstract

**Background and Purpose:**

Tranilast, in addition to its capacity to inhibit mast cell degranulation, has other biological effects, including inhibition of reactive oxygen species, cytokines, leukotrienes and prostaglandin release. In the current study, we analyzed whether tranilast could alter endothelial function in rat mesenteric resistance arteries (MRA).

**Experimental Approach:**

Acetylcholine-induced relaxation was analyzed in MRA (untreated and 1-hour tranilast treatment) from 6 month-old Wistar rats. To assess the possible participation of endothelial nitric oxide or prostanoids, acetylcholine-induced relaxation was analyzed in the presence of L-NAME or indomethacin. The participation of endothelium-derived hyperpolarizing factor (EDHF) in acetylcholine-induced response was analyzed by preincubation with TRAM-34 plus apamin or by precontraction with a high K^+^ solution. Nitric oxide (NO) and superoxide anion levels were measured, as well as vasomotor responses to NO donor DEA-NO and to large conductance calcium-activated potassium channel opener NS1619.

**Key Results:**

Acetylcholine-induced relaxation was greater in tranilast-incubated MRA. Acetylcholine-induced vasodilation was decreased by L-NAME in a similar manner in both experimental groups. Indomethacin did not modify vasodilation. Preincubation with a high K^+^ solution or TRAM-34 plus apamin reduced the vasodilation to ACh more markedly in tranilast-incubated segments. NO and superoxide anion production, and vasodilator responses to DEA-NO or NS1619 remained unmodified in the presence of tranilast.

**Conclusions and Implications:**

Tranilast increased the endothelium-dependent relaxation to acetylcholine in rat MRA. This effect is independent of the nitric oxide and cyclooxygenase pathways but involves EDHF, and is mediated by an increased role of small conductance calcium-activated K+ channels.

## Introduction

Mesenteric blood flow can constitute up to 20–30% of total cardiac output [1] and is regulated by different mechanisms in which endothelial factors like nitric oxide (NO), prostanoids and endothelium-derived hyperpolarizing factor (EDHF) play a pivotal role. Modifications in the release and/or participation of these vasoactive substances can alter peripheral vascular resistance, with the role of resistance vessels being especially relevant.

Mast cells play an important role in several physiological and pathological situations such as intestinal motility, angiogenesis and atherosclerosis [2]–[4]. When activated, mast cells secrete numerous vasoactive and proinflammatory mediators, such as histamine, serotonin, bradykinin, endothelin, NO, leukotrienes, prostaglandins, or cytokines [5], which could alter vascular endothelial and smooth muscle function [6]. These consequences are highly interesting, particularly aspects of hemodynamic changes when mast cells are stabilized. Tranilast was initially used to treat allergic diseases due to its capacity to inhibit mast cell degranulation [7] and has also been suggested in the treatment of multiple inflammatory processes, including various pathologies where blood flow is altered, such as in the vasodilation induced by allergic processes [8]–[11].

Previously our group has described that lipopolysaccharide, a model of endotoxic shock, influences vascular tone by modifying both endothelial and neuronal factors [12], [13]. Additionally**,** we have studied the effect of tranilast on the vasoconstrictor response produced by electrical field stimulation (EFS) in rat superior mesenteric arteries, demonstrating that it diminished the vasoconstrictor response to EFS by decreasing noradrenaline-induced vasoconstriction [14] although it did not influence endothelial function in this artery, as similarly reported by Yang et al [15] in rat aorta. However, mesenteric resistance arteries play a pivotal role in the regulation of vascular resistance, and differences in endothelial function have been previously described in different vascular beds under the same experimental conditions [16], [17]. With this in mind, the possible effect of tranilast on endothelial function in resistance vessels may help induce hemodynamic changes that could be relevant in the treatment of pathologies like allergy.

Since total peripheral resistance mainly depends on resistance vessels, and the role that mesenteric resistance arteries play in this is very relevant, we consider it very important to analyze the possible alterations tranilast may produce in the endothelial function of these vessels.

## Materials and Methods

### Ethics Statement

All animals were housed in the Animal Facility of the Universidad Autónoma de Madrid (Registration number EX-021U) in accordance with directive 609/86 of the E.E.C., R.D. 233/88 of the Ministerio de Agricultura, Pesca y Alimentación of Spain, and Guide for the Care and Use of Laboratory Animals published by the USA National Institutes of Health [NIH publication No. 85.23, revised 1985]. The experimental protocol was approved by the Ethics Committee of the Universidad Autónoma de Madrid.

### Animals

We used 6 month-old male Wistar rats. Rats were sacrificed by CO_2_ inhalation followed by decapitation; the mesenteric vascular bed was removed and placed in cold (4°C) Krebs-Henseleit solution (KHS; in mmol/L: 115 NaCl, 2.5 CaCl_2_, 4.6 KCl, 1.2 KH_2_PO_4_, 1.2, MgSO_4_.7H_2_O, 25 NaHCO_3_, 11.1 glucose, and 0.03 EDTA).

### Perivascular mast cell detection

The third-order branches from mesenteric resistance arteries were fixed in 4% formaldehyde in phosphate buffered saline solution (PBS, pH = 7.4) for 1 hour, cryoprotected with 30%w/v sucrose in PBS (overnight), transferred to a cryomold containing Tissue-Tek OCT embedding medium (20 min) and then immediately frozen in liquid nitrogen. All samples were kept at −70°C until the day of the experiments. Frozen tissue segments were cut into 10 µm thick sections, placed on glass slides and stained with 0.1% Toluidine Blue (3 min) for perivascular mast cell detection, as previously described [14]. Sections were coverslipped and light microscopy images were taken (Nikon Eclipse TE2000-S [inverted microscope], Nikon DXM1200F [digital camera]).

### Vascular reactivity study

For reactivity experiments the third-order branch of the mesenteric arcade was dissected and cut in segments of approximately 2 mm in length. Segments of mesenteric resistance arteries were mounted in a small vessel chamber myograph (Danish Myo Technology A/S, Äarhus, Denmark) to measure isometric tension according to the method described by Mulvany and Halpern [18]. After a 15-min equilibration period in oxygenated KHS at 37°C and pH 7.4, segments were stretched to their optimal lumen diameter for active tension development. Optimal lumen diameter was determined based on the internal circumference/wall tension ratio of the segments by setting the internal circumference, L_ 0_, to 90% of what the vessels would have if they were exposed to a passive tension equivalent to that produced by a transmural pressure of 100 mmHg [18]. Optimal lumen diameter was determined using specific software for normalization of resistance arteries (DMT Normalization Module; ADInstruments Pty Ltd, Castle Hill, Australia). Segments were washed with KHS and left to equilibrate for 30 min. Vessel contractility was then tested by an initial exposure to a high-K^+^ (120 mmol/L) solution.

After washout, segments were contracted with a concentration of noradrenaline that induced approximately 50%–70% of the maximum contraction elicited by KCl, and then acetylcholine (1 µmol/L) was added to assess the integrity of the endothelium. Some segments were subjected to mechanical endothelium removal. The absence of endothelium was confirmed by the inability of acetylcholine (1 µmol/L) to induce relaxation. Endothelium removal did not modify KCl- (120 mmol/L) induced contraction.

Since the level of smooth muscle constriction can itself antagonize the extent of the endothelium-dependent relaxation, we performed the following experiments adjusting the dose of NA or KCl to a concentration which allowed us to reach a 50–70% of the maximum contraction elicited by KCl.

### Experimental protocols

The segments were rinsed with KHS for 1 h and then a cumulative concentration-response curve to ACh (0.1 nmol/L to 3 µmol/L) was obtained in noradrenaline-precontracted segments preincubated or not with tranilast (100 µmol/L, 1 hour, time and dose obtained from previous pilot studies). The concentration of tranilast used and the time of incubation were from previous pilot studies, performed similarly to our previous study [14]. Additionally, vasoconstrictor responses to alpha-adrenergic agonist noradrenaline (10 nmol/L to 0.1 mmol/L) were performed in both control and tranilast-incubated segments.

The possible role of NO in ACh-induced relaxation was investigated in tranilast-treated and untreated segments by preincubation with 100 µmol/L L-NAME (a non-selective nitric oxide synthesis inhibitor) before performing concentration-response curves to ACh. Additionally, endothelium-independent relaxation was studied by evaluating relaxation to NO donor DEA-NO (10 nmol/L to 300 µmol/L) in arteries previously contracted with noradrenaline.

The role of EDHF in the ACh-induced relaxation was analyzed. For this purpose, the vasodilator response to ACh in segments precontracted high K^+^ solution (at a concentration that produced approximately 50–70% of the contraction induced by 120 mM KCl) was studied. Additionally, the effect of a calcium-activated potassium channel blockade, produced by apamin (1 µmol/L) plus TRAM-34 (0.1 µmol/L), on the ACh response was analyzed in NA-precontracted arteries pretreated or not with tranilast. In another set of experiments, the effect of L-NAME plus TRAM-34 plus apamin on ACh-induced relaxation was studied. To determine whether tranilast modified the participation of each potassium channel individually, concentration response curves to acetylcholine were performed in the presence of L-NAME plus apamin or L-NAME plus TRAM-34. All drugs were added 30 min before the concentration-response curve to ACh. Additionally, to rule out an effect of tranilast on NO mediated hyperpolarization, concentration-response curves to DEA-NO were performed in control and tranilast-incubated mesenteric segments precontracted with a high K^+^ solution.

The effect of tranilast on the smooth muscle calcium-activated potassium channels was analyzed. For this purpose, the relaxation produced by NS1619 (10 nmol/L-100 µmol/L), a large conductance calcium-activated potassium channel opener, was analyzed in NA-precontracted endothelium-denuded arteries preincubated or not with tranilast.

The participation of COX-derived metabolites was investigated in tranilast-treated and untreated segments. Arteries were preincubated with the non-specific COX inhibitor indomethacin (10 µmol/L) before performing concentration-response curves to ACh.

### Nitric Oxide release

Nitric oxide release was determined using the fluorescent probe 4,5-diaminofluorescein (DAF-2), as previously described [19]. Briefly, the second, third and fourth branches of mesenteric artery were divided in two experimental groups: control and tranilast-incubated segments (100 µmol/L, 1 hour). After an equilibration period of 30 min in HEPES (in mmol/L: 119 NaCl, 20 HEPES, 46 KCl, 1 MgSO_4_.7H_2_O, 0.15 Na_2_HPO_4_.12H_2_O, 0.4 KH_2_PO_4_, 5 NaHCO_3_, 1.2 CaCl_2_.2H_2_O, 5.2 glucose) at 37°C, arteries were incubated with 2 µmol/L DAF-2 for 45 min and medium was collected to measure basal NO release. Once the organ bath was refilled, ACh-induced NO release was measured after an ACh concentration-curve (0.1 nmol/L - 3 µmol/L) was applied at 2-min intervals each dose. The fluorescence of the medium was measured at room temperature using a spectrofluorimeter (LS50 Perkin Elmer Instruments, FL WINLAB Software) with excitation wavelength set at 492 nm and emission wavelength at 515 nm. The stimulated NO release was calculated by subtracting the basal NO release from that evoked by ACh. Also, blank measurement samples were collected from medium without mesenteric segments in order to subtract background emission. Some assays were performed in the presence of L-NAME in order to assure assay specificity. The amount of NO released was expressed as arbitrary units/mg tissue.

### Detection of superoxide anions

Superoxide anions levels were measured using lucigenin chemiluminescence, as previously described [20]. Briefly, the second, third and fourth branches of mesenteric artery, divided in two experimental groups, control and tranilast-incubated segments (100 µmol/L, 1 hour), were equilibrated for 30 min in HEPES buffer at 37°C, transferred to test tubes that contained 1 mL HEPES buffer (pH 7.4) containing lucigenin (5 µmol/L) and then kept at 37°C. The luminometer was set to report arbitrary units of emitted light; repeated measurements were collected during 5 min at 10 s intervals and averaged. 4,5-dihydroxy-1,3-benzene-disulphonic acid ‘‘Tiron’’ (10 mmol/L), a cell permeant, non-enzymatic superoxide anion scavenger, was added to quench the superoxide anion-dependent chemiluminescence. Also, blank samples were collected in the same way without mesenteric segments to subtract background emission.

### Drugs

Drugs used were tranilast, atropine, noradrenaline hydrochloride, acetylcholine chloride, DEA-NO, indomethacin, apamin, tiron, TRAM-34, NS1619 (Sigma; St. Louis, MO, U.S.A.). Stock solutions of acetylcholine, apamin, tiron, TRAM-34 and DEA-NO were made in distilled water, noradrenaline was dissolved in a NaCl (0.9%)-ascorbic acid (0.01% wv-1) solution; indomethacin was dissolved in ethanol;tranilast, NS1619 was dissolved in dimethyl sulfoxide. These solutions were kept at −20°C and appropriate dilutions were made on the day of the experiment.

### Statistical analysis

Contractions to noradrenaline were expressed as the percentage of contraction of the maximum contractile response induced by a previous contraction of KCl. Relaxation to ACh, DEA-NO and NS1619 were expressed as a percentage of the level of preconstriction induced by noradrenaline or KCl. For each concentration-response curve the maximum effect (Emax) and the concentration of agonist that produced half of the Emax (log EC50) were calculated using non-linear regression analysis (GraphPad Prism Software, San Diego, CA). The sensitivity of the agonists is expressed as pD2 (−log EC50).

All values are expressed as means ± S.E.M. of the number of animals used in each experiment. Statistical analysis was done by comparing the curve obtained in the presence of the different substances with the control curve by means of a non-repeated measure analysis of variance (ANOVA) followed by the Bonferroni post-hoc test. Some results were expressed as differences of area under the curve (dAUC). AUC were calculated from the individual concentration-response plots. For dAUC, NO and superoxide anion release experiments, the statistical analysis was done using one-way ANOVA followed by Newman-Keuls post-hoc test. P<0.05 was considered significant.

## Results

**Figure 1:**
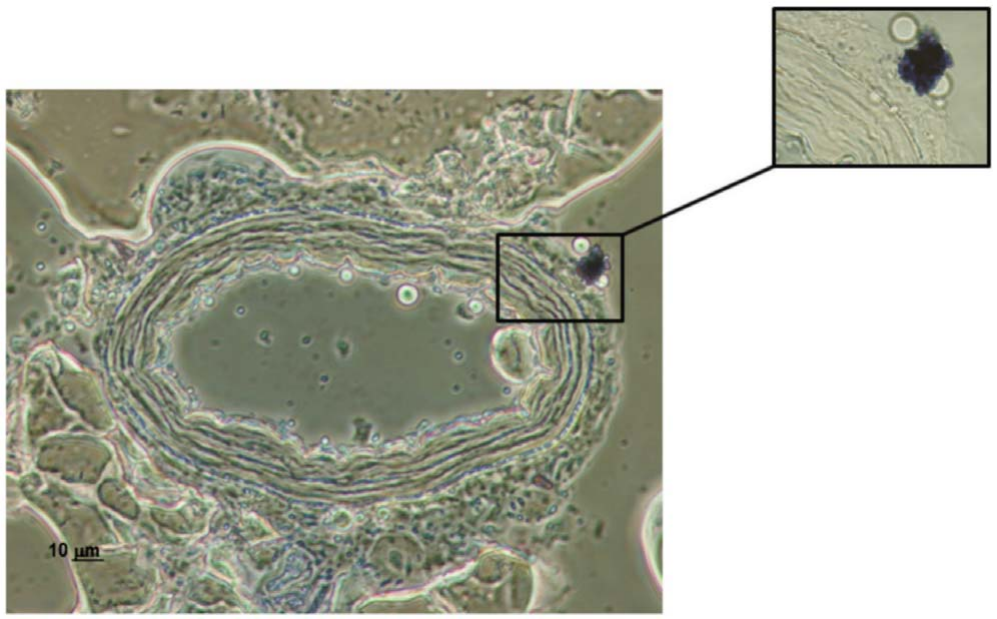
Mast cell localization by toluidine blue staining. Figure is representative of preparations from four rats. Magnification: 400X (general vision) and 600X (inset).

Mast cells were detected in the adventitial layer of mesenteric arteries using toluidine blue staining (Figure 1).

**Figure 2:**
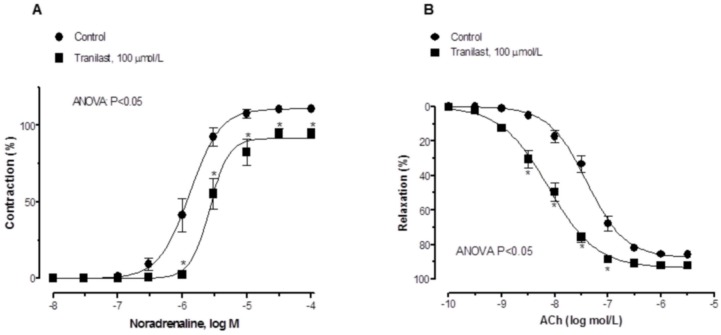
Effect of tranilast on endothelial function. NA-induced vasoconstriction in control and tranilast-treated mesenteric resistance arteries (A).Endothelium-dependent relaxation induced by ACh in NA-precontracted control and tranilast-treated rat resistance arteries (B) Results are expressed as mean ± S.E.M. *P<0.05 control vs. tranilast. N = 6–7 animals each group.

Preincubation with 100 µmol/L tranilast did not modify vasoconstrictor response to 120 mmol/L KCl (Control: 14.5±1.5 mN; Tranilast: 15.1±1.3 mN/mm; p>0.05), while it shifted the noradrenaline-induced contractile curve to the right (Figure 2A). Cumulative addition of ACh evoked endothelium-dependent relaxations in noradrenaline-contracted arteries. 10 µmol/L and 1 µmol/L tranilast concentrations did not produce any modification on ACh-induced vasodilation in 1–3 hours incubations (Results not shown), while 1 hour-preincubation with 100 µmol/L tranilast shifted the concentration response curve to ACh to the left (Figure 2B and Table 1).

**Table 1:** Effect of indomethacin, L-NAME, Apamin plus TRAM-34 or L-NAME plus Apamin plus TRAM-34 on E_max_ and pD2 to acetylcholine in untreated and tranilast-treated MRA.

	Untreated			Tranilast-treated	
	E_max_	pD_2_	E_max_		pD_2_
Control	87.6±2.03	7.43±0.04	93.5±1.64		8.12±0.04+
Indomethacin	85.5±2.51	7.44±0.06	97.1±5.83		8.11±0.13+
L-NAME	71.4±3.09*	7.27±0.07	87.1±2.18+		7.69±0.11* +
Apamin+TRAM-34	43.5±5.55*	7.29±0.12	46.6.5±2.82*		7.47±0.11*
L-NAME+Apamin+TRAM-34	4.33±2.60*	-	3.83±2.21*		-

Values represent means ± S.E.M.

*P<0.05 *vs*. situation without specific drugs;

+ P<0.05, Tranilast-treated *vs*. untreated.

**Figure 3:**
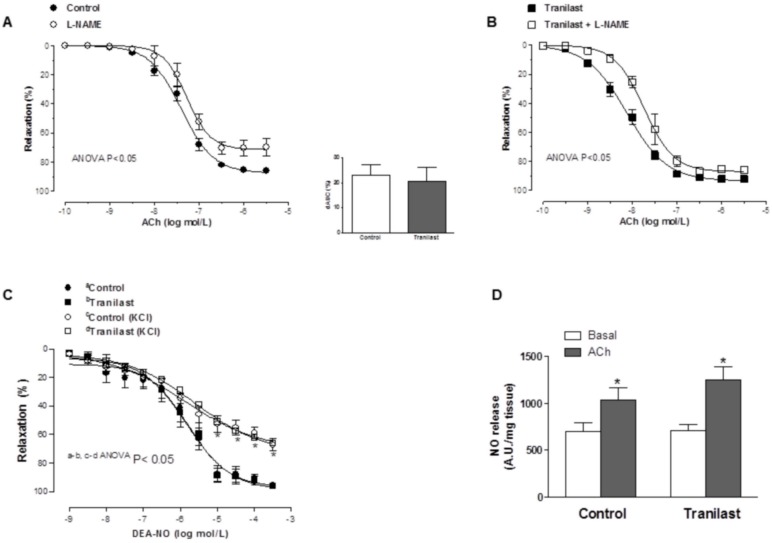
Participation of NO on the vasodilator response to acetylcholine. Effect of L-NAME (100 µM) on the concentration-dependent relaxation to ACh in control (A) and tranilast-treated (B) mesenteric resistance arteries. Insert graph shows the differences of area under the curve (dAUC) in control and tranilast-treated arteries pre-treated with L-NAME. Results are expressed as mean ± SEM. N = 6–7 animals in each group. (C) Vasodilator response to DEA-NO in control and tranilast-incubated mesenteric resistance arteries, precontracted with either noradrenaline or KCl. Results are expressed as mean ± S.E.M. N = 5–6 animals each group. (D) Effect of tranilast on basal and acetylcholine-induced NO release in rat mesenteric resistance arteries. Results (mean ± S.E.M.) are expressed as arbitrary fluorescence units (A.U.)/mg tissue. N = 4 animals each group. *P<0.05 vs. basal.

NO synthase inhibitor L-NAME decreased ACh-induced relaxation to a similar extent in both control and tranilast-incubated mesenteric segments (Figures 3A and 3B, Table 1). Relaxation to DEA-NO was not changed by tranilast either in NA-precontracted or in KCl-precontracted mesenteric arteries (Figures 3C, Table 2). In line with this, both basal and ACh-stimulated NO releases were similar in tranilast-treated and untreated mesenteric resistance arteries (Figure 3D). Preincubation with L-NAME abolished NO release in all experimental groups (results not shown). Superoxide anion release was similar in both tranilast-treated and untreated segments (In chemiluminiscence units/min mg tissue: Control: 10.92±3.5; Tranilast: 12.03±3.7; P<0.05.).

**Table 2:** E_max_ and pD2 values of DEA-NO in untreated and tranilast-treated MRA.

	Untreated		Tranilast-treated	
	E_max_	pD_2_	E_max_	pD_2_
NA-precontracted	96.95±5.12	5.78±0.13	94.25±4.46	5.73±0.10
KCl-precontracted	70.89±3.31*	5.87±0.23	72.97±4.07*	5.61±0.12

Values represent means ± S.E.M.

*P<0.05 KCl precontraction *vs*. NA precontraction.

*P<0.05 NA-precontracted vs. KCl precontracted.

**Figure 4:**
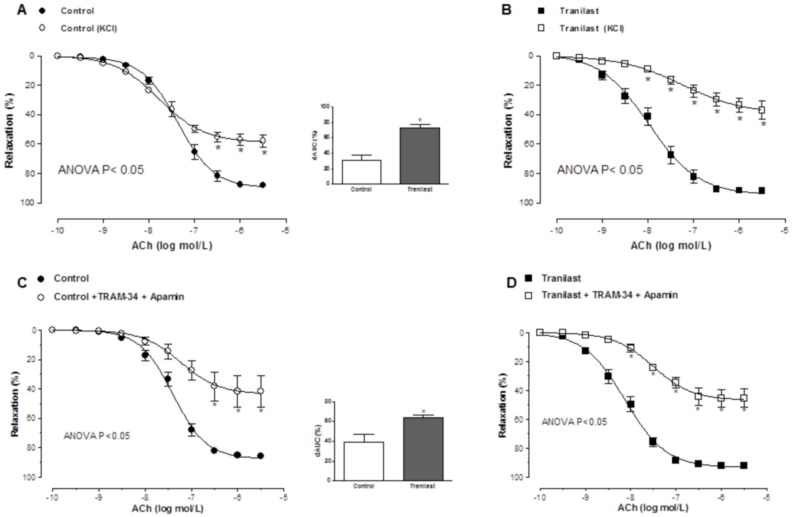
Participation of EDHF in the vasodilator response to acetylcholine. Relaxation to acetylcholine in control (A) and tranilast-treated arteries (B) pre-contracted with KCl. Effect of preincubation with 1 µM apamin plus 0.1 µM TRAM-34 on endothelium-dependent relaxation to acetylcholine in noradrenaline-pre-contracted control (C) and tranilast-treated arteries (D). Insert graph shows the differences of area under the curve (dAUC) in control and tranilast-treated arteries either preconstricted with KCl or pre-treated with TRAM-34 plus Apamin. Results are expressed as mean ± SEM. *P<0.05 control vs. tranilast. N = 5–7 animals in each group.

**Figure 5:**
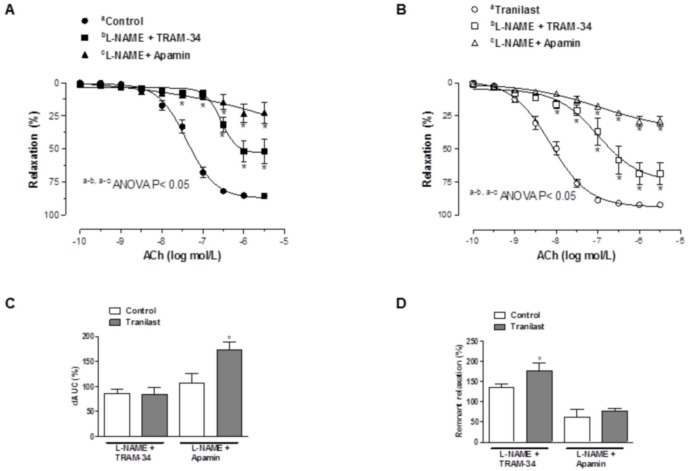
Participation of potassium channels in the vasodilator response to acetylcholine. Effect of preincubation with 100 µmol/L L-NAME plus 1 µM apamin or plus 0.1 µM TRAM-34 on endothelium-dependent relaxation to acetylcholine in noradrenaline-pre-contracted control (A) and tranilast-treated arteries (B). Results are expressed as mean ± SEM. *P<0.05 control vs. tranilast N = 5–7 animals in each group. (C) Differences of area under curve (dAUC) in the absence or presence of 100 µmol/L L-NAME plus 1 µM apamin or plus 0.1 µM TRAM-34. Results are expressed as mean ± SEM. dAUC values are expressed as percentage. *P<0.05 control vs. tranilast. N = 5–7 animals each group. (D) Representation of remnant acetylcholine-induced vasodilation after preincubation with 100 µmol/L L-NAME plus 1 µmol/L apamin or plus 0.1 µM TRAM-34, expressed as mean ± SEM of percentage of AUC. *P<0.05 control vs. tranilast. N = 5–7 animals each group.

**Figure 6:**
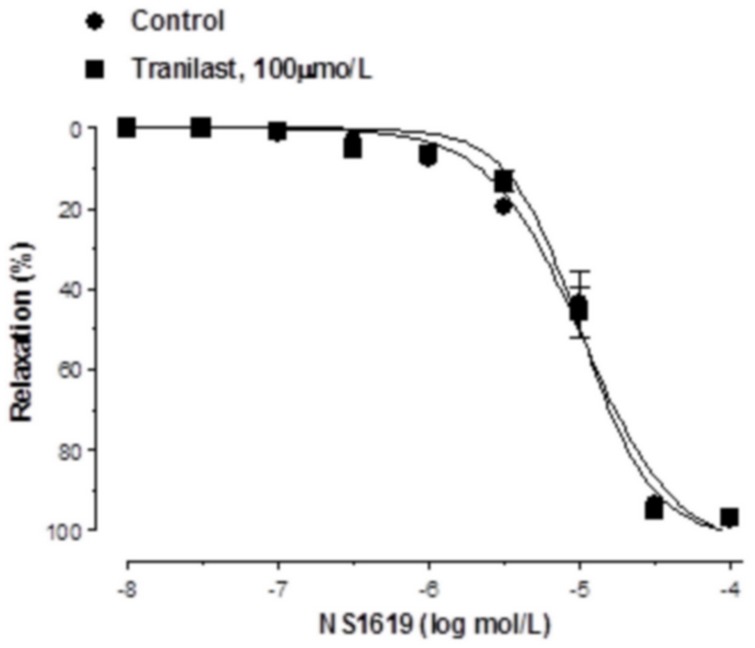
Vasodilator response to K^+^-channel openers. Effect of tranilast on the relaxation to the large conductance calcium-activated K^+^-channel opener NS1619 in de-endothelized rat mesenteric arteries. Results are expressed as mean ± SEM. N = 5–7 animals in each group.

The concentration response curve to ACh was shifted to the right in KCl-precontracted segments after preincubation with 100 µmol/L tranilast (Figure 4A and 4B). Similarly, preincubation with apamin plus TRAM-34 shifted the ACh-induced relaxation leftward to a greater extent in tranilast-incubated segments than in control segments (Figure 4C and 4D). Combined preincubation with L-NAME plus TRAM-34 reduced ACh-induced relaxation similarly in both control and tranilast-incubated segments. However, preincubation with both L-NAME and apamin shifted the ACh-induced relaxation to the left more markedly in tranilast-incubated segments. The remnant vasodilation observed after preincubation with L-NAME plus TRAM-34 was higher in tranilast-incubated compared to control segments, while it was similar in both experimental conditions after preincubation with L-NAME plus apamin. (Figure 5). Vasodilator response to NS1619 remained unmodified in presence of tranilast. (Figure 6).

**Figure 7:**
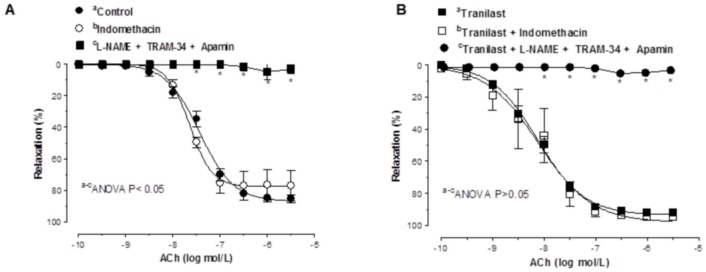
Participation of prostanoids in the vasodilator response to acetylcholine. Effect of preincubation with 10 µM indomethacin or with 100 mmol/L L-NAME plus 1 µM apamin plus 0.1 µM TRAM-34 on the concentration-dependent relaxation to ACh in control (A) and tranilast-treated (B) rat mesenteric resistance arteries. Results are expressed as mean ± S.E.M. *P<0.05 control vs. tranilast. N = 6–7 animals in each group.

In tranilast-treated and untreated segments, ACh-induced vasodilation was not modified by indomethacin (Figure 7, Table 1). In line with this, the combined inhibition of NO and EDHF through preincubation with L-NAME plus apamin plus TRAM-34 abolished the increase in relaxation to ACh produced by tranilast (Figure 7, Table 1).

## Discussion

The present results show that tranilast increased the endothelium-dependent relaxation to ACh in rat mesenteric resistance arteries. This effect is independent of the NO or COX pathways and seems to be mediated by an increase in EDHF contribution.

Under physiological conditions, mast cells have been identified in several locations in the mesentery, including around the mesenteric vessels [14], [21]. When activated, mast cells secrete numerous vasoactive and proinflammatory mediators, such as histamine, serotonin, bradykinin, endothelin, NO, leukotrienes, prostaglandins, or cytokines [5], which could alter vascular endothelial and smooth muscle function [22]. Tranilast is a mast cell stabilizer used in various pathologies where blood flow is altered [8]–[11], such as allergy, which produces an intense vasodilation produced by histamine release from mast cells. In this study we have located perivascular mast cells around mesenteric resistance vessels, as has been described in superior mesenteric artery [14]. Previously, we have described that tranilast decreases EFS-induced vasoconstriction in superior mesenteric arteries [14]. Since total peripheral resistance mainly depends on resistance vessels, and the role that mesenteric resistance arteries play in this is very relevant, we consider it very important to analyze the possible alterations tranilast may produce in the endothelial function of these vessels. When analyzing endothelium-dependent relaxation induced by ACh in mesenteric resistance arteries, we observed an increase in this vasodilator response in segments preincubated with tranilast. Similar changes in the endothelial function observed in several pathologic situations in these vessels are associated to decreased vascular resistance and subsequent hemodynamic changes [19], [23]. This outcome contrasts with previous studies, in which ACh-induced vasodilation was not modified by tranilast in superior mesenteric artery or aorta, despite the longer treatment period used [14], [15]. These results indicate that tranilast can modify endothelial factor release and/or sensitivity differentially depending on the vascular bed analyzed, which is not surprising since we have previously described a similar effect in these vascular beds [24], probably associated to differences in the composition of endothelial factors.

Previous studies show that endothelial dysfunction is related to an increase in the vasoconstrictor responses to different agonists and vice versa [20], [25], [26]. In our experimental conditions, vasoconstriction produced by KCl remained unmodified in the presence of tranilast, similar to observations in superior mesenteric arteries [14], suggesting that this drug does not modify vascular contractile capacity. Additionally, when analyzing the vasoconstrictor response to the alpha-adrenergic agonist noradrenaline, we observed that response was decreased in tranilast-incubated segments, similarly to descriptions in superior mesenteric artery [14], but in contrast to observations in rat aorta [15].

The relaxation evoked by ACh is mediated, depending on the vascular bed analyzed, by the release of endothelium-dependent relaxing factors such as NO, prostacyclin, and EDHF [27]–[29]. In rat mesenteric resistance arteries this relaxation is mainly mediated by the release of NO and EDHF [30], but not by COX-derived products [31]. Contradictory effects of tranilast on NO release have been described, since both increases [32], decreases [14], [33], [34] and no modifications [15] of NO release have been reported in several tissues after tranilast preincubation. Additionally, multiple studies have described an antioxidant effect of tranilast treatment in both *in vivo* and *in vitro* experimental procedures [14], [32], [33], [35]–[39]. With this in mind, the effects produced by tranilast in ACh-induced vasodilation could be mediated by changes in NO synthesis and/or bioavailability. In order to analyze this possibility, we preincubated control and tranilast-exposed mesenteric resistance segments with the non-specific NOS inhibitor L-NAME. We observed that, after preincubation with this drug, ACh-induced relaxation was decreased to a similar extent in both experimental conditions indicating that NO does not participate in the effect observed after preincubation with tranilast. This was confirmed by the fact that NO release, superoxide anion formation and vasodilator response to NO donor DEA-NO were not modified after preincubation with tranilast, similarly to reported in rat aorta [15]. All these results contrast with our previous results in superior mesenteric artery, where we observed decreases in neuronal NO and superoxide anion releases and an increase in the vasodilator response to DEA-NO after tranilast preincubation [14]. In conclusion, the results obtained in the present study confirm the fact that the increased vasodilator response to ACh produced by tranilast is not due to modifications in the NO pathway.

Hyperpolarizing mechanisms are important regulators of the membrane potential and hence of vessel tone [29], this mechanism being particularly important in small arteries and arterioles. Although controversial, NO has been described to exert a hyperpolarizing role in several vascular beds [40], [41]. This hyperpolarization produced by NO can be due to an activation of different potassium channels, including large-conductance calcium dependent potassium channels and voltage-dependent potassium channels [42]. Thus, the effects of tranilast on non-membrane potential-dependent actions of DEA-NO were investigated in arteries preconstricted with a high K^+^ solution, thus blocking hyperpolarization by decreasing the plasma membrane potassium gradient [43]. The results showed that, in KCl-precontracted arteries, the vasodilator response induced by DEA-NO was reduced to a similar extent in control and tranilast-incubated mesenteric segments, confirming the hyperpolarizing role of NO in this vascular bed, and also that this effect is not altered by tranilast.

EDHF plays, in addition to NO, an important vasodilator role in resistance vessels. The relaxation induced by EDHF is endothelium-dependent, insensitive to inhibition by a combination of NOS and COX inhibitors, and leads to hyperpolarization of vascular smooth muscle cells [44]. In order to determine whether the increase in ACh-induced vasodilation induced by tranilast is due to an increase in EDHF participation, control and tranilast-incubated mesenteric resistance arteries were precontracted with a high K^+^ solution. We observed that, in this experimental condition, vasodilation to ACh was reduced in both control and tranilast-incubated segments, but more markedly in segments exposed to tranilast. Initially, the EDHF-mediated response was attributed to activation of small, intermediate and large conductance calcium-activated K^+^-channels, although the participation of the latter has been questioned [45]–[49]. In presence of a combination of small (SKCa) and intermediate conductance calcium-activated K^+^-channel (IKCa) blockers (apamin+TRAM-34, respectively), we also observed a greater inhibition of the ACh-induced vasodilation in tranilast-preincubated segments compared to control segments. However, a differential effect of tranilast on each type of calcium-activated potassium channel must also be considered. The fact that a combined preincubation with L-NAME plus TRAM-34 decreased ACh-induced relaxation similarly in both experimental conditions, while a combination of L-NAME plus apamin produced a more marked decrease in ACh-induced relaxation in tranilast-incubated segments, suggests a greater participation of SKCa channels through tranilast preincubation. These findings indicate that hyperpolarization produced by EDHF is responsible for the tranilast-mediated effects on the ACh-induced dilation in mesenteric resistance arteries, due to an increased SKCa channel participation after preincubation with tranilast. Since the importance of the hyperpolarizing mechanism in endothelium-dependent relaxations increases as the vessel size decreases [50], [51], this result can explain the difference in the effect of tranilast on ACh-induced vasodilation previously observed in superior mesenteric artery and aorta, where the role of EDHF in endothelium-dependent relaxation is essentially absent [14], [15].

The greater participation of EDHF in ACh-induced response in tranilast-incubated arteries may be associated to an increase in potassium channel activation by EDHF or to an increase in EDHF generation. The fact that the vasodilation induced by NS1619 (a large conductance calcium-activated K^+^-channel opener) was not altered in the presence of tranilast seems to rule out a greater activation of these channels by the tranilast effect. However, we must take into account that these channels are also present in endothelial cells, whose activation alters the release of several vasoactive substances [52]–[56]. Taken together our results indicate that tranilast increases the vasodilator response to ACh through a mechanism that implicates a greater participation of EDHF. This effect seems to be associated with a greater activation of SKCa channels, without modifying the participation of IKCa channels,

As we have previously reported [31], COX-derived products do not participate in the relaxation induced by ACh in control situations in mesenteric resistance arteries. However, in some pathological situations, such as hyperaldosteronism, we have also described participation by COX-derived products in vascular function, including relaxation to ACh [19], [31]. In the present study, the COX inhibitor indomethacin did not affect the relaxation to ACh in the absence or presence of tranilast, confirming the non-participation of COX-derived products in both experimental conditions. The fact that in the presence of L-NAME plus TRAM-34 plus apamin the relaxation to ACh was abolished confirmed this observation, since it demonstrates that the vasodilator response to ACh is only due to NO and EDHF in these experimental conditions.

In summary, tranilast increased the endothelium-dependent relaxation to acetylcholine in rat mesenteric resistance arteries. This effect is independent of the NO and COX pathways but involves EDHF, and is mediated by an increased role of small conductance calcium-activated K^+^ channels. Similar alterations in endothelial function in this vascular bed have been associated to altered splanchnic circulation and the development of organ failure [19]. Therefore, these results lead us to consider it important to evaluate the hemodynamic conditions of patients receiving treatment with tranilast.
